# Supplementary material for: Splice-Junction-Based Mapping of Alternative Isoforms in the Human Proteome
Source: Cell Rep. Author manuscript; Available in PMC 2020 Jan 15. (PMC6961840; doi:10.1016/j.celrep.2019.11.026)
Supplement: 3 [file NIHMS1546469-supplement-3.zip › DF2/PXD006675/LeftVentricle-83-O43314-TATNDVVSENANYLR.pdf]

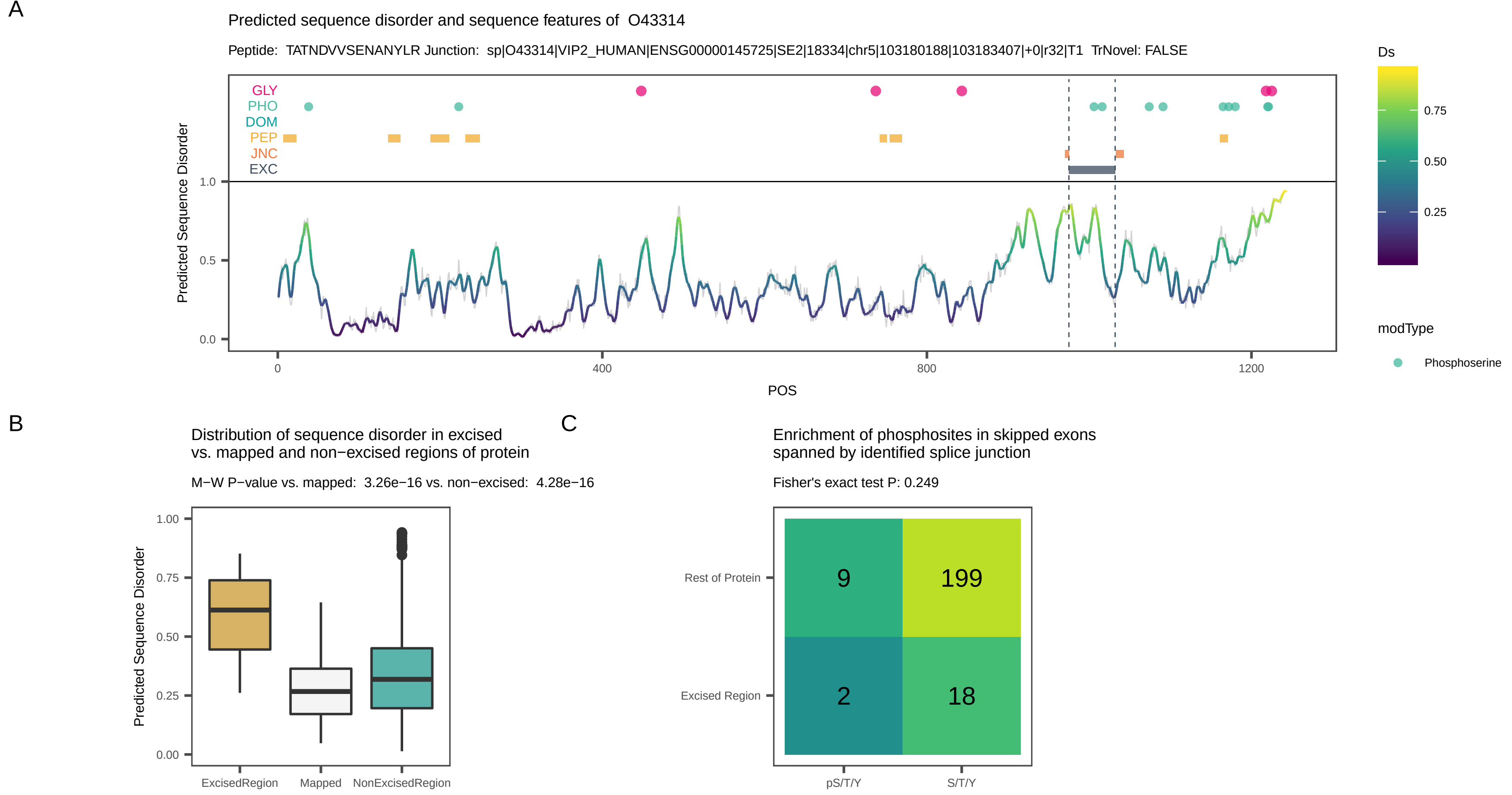

A
Predicted sequence disorder and sequence features of O43314
Peptide: TATNDVVSENANYLR Junction: sp|O43314|VIP2_HUMAN|ENSG00000145725|SE2|18334|chr5|103180188|103183407|+0|r32|T1 TrNovel: FALSE
GLY
PHO
DOM
PEP
JNC
EXC
Predicted Sequence Disorder
1.0
0.5
0.0
0
400
800
1200
POS
Ds
0.75
0.50
0.25
modType
Phosphoserine
B
Distribution of sequence disorder in excised
vs. mapped and non−excised regions of protein
M−W P−value vs. mapped: 3.26e−16 vs. non−excised: 4.28e−16
Predicted Sequence Disorder
1.00
0.75
0.50
0.25
0.00
ExcisedRegion
Mapped
NonExcisedRegion
C
Enrichment of phosphosites in skipped exons
spanned by identified splice junction
Fisher's exact test P: 0.249
Rest of Protein
Excised Region
9
199
2
18
pS/T/Y
S/T/Y
